# A dose-independent association of triglyceride levels with all-cause mortality among adults population

**DOI:** 10.1186/s12944-020-01400-w

**Published:** 2020-10-15

**Authors:** Yu-qing Huang, Xiao-cong Liu, Kenneth Lo, Ying-qing Feng, Bin Zhang

**Affiliations:** 1 Department of Cardiology, Guangdong Cardiovascular Institute, Guangdong Provincial People’s Hospital, Guangdong Academy of Medical Sciences, South China University of Technology School of Medicine, No. 106, Zhongshan Second Road, Yuexiu District, Guangzhou, 510080 China; 2 Centre for Global Cardiometabolic Health, Department of Epidemiology, Brown University, Providence, RI USA; 3 Department of Applied Biology and Chemical Technology, The Hong Kong Polytechnic University, Hung Hom, Hong Kong, China

**Keywords:** Triglyceride, All-cause mortality, Cardiovascular mortality, Adult population, Nonlinear, Dose-independent

## Abstract

**Background:**

The relationship between triglyceride (TG) level and the mortality risk of all-cause and cardiovascular disease is not entirely consistent among adults.

**Methods:**

The present analysis included adult participants from National Health and Nutrition Examination Surveys (NHANES) between the periods 1999–2014. The levels of TG were categorized into < 150, 150–199, 200–250 and ≥ 250 mg/dL respectively. Multivariate Cox regression analysis, stratified analysis and generalized additive model were conducted to reveal the correlation between TG and mortality risk. Results were presented in hazard ratio (HRs) and 95% confidence intervals (CIs).

**Results:**

There were 18,781 (9130 males, mean age was 45.64 years) participants being included in the analysis. The average follow-up period was 8.25 years, where 1992 (10.61%) cases of all-cause and 421 (2.24%) cardiovascular death have occurred. In the multivariate Cox model, every 1 mg/dL raise in TG has significantly associated with all-cause mortality (HR: 1.08, 95% CI: 1.02, 1.15) but not cardiovascular mortality (HR: 1.10, 95% CI: 0.97, 1.24). When using TG <  150 mg/dL as reference, TG ≥ 250 mg/dL associated with death from all-cause (HR = 1.34, 95% CI: 1.12, 1.60; *P* = 0.0016 but not cardiovascular death (HR = 1.26, 95% CI: 0.85, 1.88; *P* = 0.2517). According to smoothing spline plots, the risk of all-cause was the lowest when TG was approximately 135 mg/dL.

**Conclusion:**

TG might have a dose-independent association with all-cause mortality among adults in United States.

## Introduction

Multiple epidemiological and clinical studies have reported the linkage between elevated triglyceride (TG) concentrations and cardiovascular diseases (CVD) [1–5]. The Bezafibrate Infarction Prevention Registry study revealed that elevated TG associated with higher mortality risk in patients with coronary heart disease [6]. More recently, a meta-analysis of 61 cohorts demonstrated relationship between TG levels and CVD in a dose-response manner [7]. Another meta-regression analysis of 49 randomized trials has found that lowering TG could reduce the risk of major vascular events, which was independent from the levels of circulating low density lipoprotein cholesterol (LDL-C) [8]. However, the relation between TG and mortality was often attenuated after being adjusting for cardiovascular risk factors. Currently, whether triglyceride concentration was independently related to all-cause or cause-specific mortality has been controversial. Davis, et al. [9] suggested that the levels of TG had no independent association with mortality due to coronary disease. Khawaja et al. [10] demonstrated that lower TG (TG < 200 mg/dL) at admission was associated with a higher risk of mortality in participants with myocardial infarction. Considering the discrepancies in findings, the prospective relationship between serum TG levels and mortality was explored among adults in United States (US).

## Methods

### Subjects enrollment

**Fig. 1:**
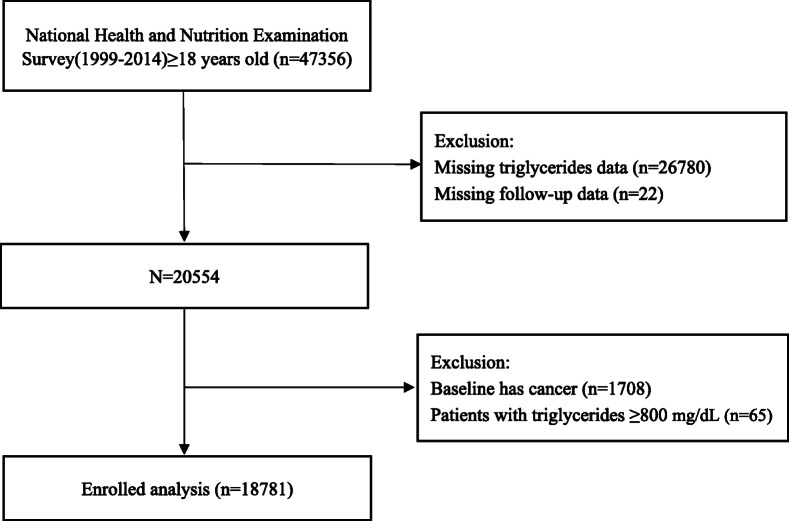
Research flow chart

The data source was from National Health and Nutrition Examination Surveys (NHANES) between the periods 1999–2014. NHANES was a nationwide study conducted by Centers for Disease Control and Prevention (CDC) in United States. Serum TG was determined among NHANES participants aged ≥18 years old. After excluding participants younger than 18, people with missing serum TG levels or with cancer at baseline, 18,781 participants were included (Fig. 1). The Institutional Review Board of the CDC has approved the protocol of survey (Protocol 98–12, 2005–06 and 2011–17). Participants provided informed consent in written form before the start of the study.

### Assessment of exposure

Standardized procedures and methods were used for collecting serum samples. Serum samples were collected from venous vessels in the morning. Serum circulating TG and total cholesterol (TC) levels were measured enzymatically, while direct immunoassay or precipitation was used to determine the level of high-density lipoprotein cholesterol (HDL-C) [11]. Hitachi 704 Analyzer (Boehringer Mannheim Diagnostics, Indianapolis, IN) was used to measure serum TC, HDL-C and TG [12]. If TG was ≤400 mg/dL, Friedewald formula was adapted to calculate the value of LDL-C [13].

### Assessment of covariates

At baseline, socio-demographics and lifestyle factors (including age, race/ethnicity, gender, smoking status, diet habits and education levels), data on physical examination (height, weight, blood pressure, estimated glomerular filtration rate (eGFR)), disease history and medication history were acquired. Hypertension was defined by self-reported history, systolic/diastolic blood pressure (SBP/DBP) ≥ 140/90 mmHg, or taking drugs to reduce blood pressure [14]. Diabetes was defined by self-reported history, taking hypoglycemic medications, fasting serum glucose level ≥ 7.0 mmol/L or hemoglobin A1C ≥ 6.5% [15].

### Assessment of outcome

The outcomes of this study were death from all-cause and CVD. Mortality status was ascertained from NHANES until death or 31st December 2015, whichever occurred first. Codes in the 10th edition of the International Classification of Diseases including I00-I09, I20-I51, I11 and I13 were used to derive the cause of CVD death [16].

### Statistical analyses

Descriptive statistics was presented according to the levels of TG (< 150, 150–200, 200–249, ≥ 250 mg/dL) at baseline. Differences by TG levels were explored by one-way analysis of variance, Kruscal Whallis *H* test and chi-square tests. Multivariate Cox regression models with hazard ratios (HRs) and 95% confidence intervals (CIs) were built to assess the death from all-cause and CVD. Model I was crude model and no confounders were included. In Model II, age, sex and body mass index (BMI) were adjusted. In Model III, race, level of education, smoking status, SBP, energy intake, C-reactive protein, eGFR, TC, HDL-C, disease history and the use of medication were additionally adjusted. Differences in survival rates by TG levels were analyzed by Kaplan-Meier curves. Restricted cubic spline analysis was used to reveal how serum TG might relate to all-cause and CVD mortality. A generalized additive model was used to assess any nonlinear relationship. If a nonlinear relationship was detected, Cox proportional hazards models were built on both sides of the inflection point. Results from Cox regression were stratified by age at baseline (< 65 or ≥ 65 years), sex (man or woman), ethnicity (White or non-White), BMI categories (< 25 or ≥ 25 kg/m^2^), history of diabetes or hypertension, history of CVD and the use of lipid-lowering drugs (all categorized into yes or no). Statistical analyses were performed by R software with the version of 3.3.2 (Vienna, Austria) and *P* <  0.05 was regarded as statistically significant.

## Results

### Baseline characteristics

**Fig. 2:**
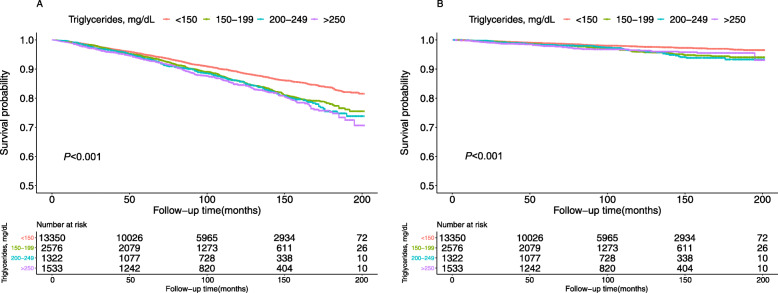
Kaplan-Meier survival curves for all-cause (**a**) and cardiovascular (**b**) mortality

The present study included 18,781 participants (9130 males, mean age was 45.64 years). During the average follow-up period of 8.25 years, 1992 (10.61%) all-cause and 421 (2.24%) cardiovascular death occurred. Table 1 has summarized the characteristics of participants at baseline. There were significant subgroup differences for all baseline variables according to TG levels. The differences in survival rate of all-cause (Fig. 2a) and cardiovascular (Fig. 2b) mortality according to the levels of TG were demonstrated in Fig. 2.

**Table 1:** Demographic and clinical characteristics according to triglyceride levels

Characteristic		Triglycerides, mg/dL				*P*-value
	Total	< 150	150–199	200–250	≥ 250	
Number	18,781	13,350	2576	1322	1533	
Age, years	45.64 ± 18.94	44.30 ± 19.15	49.09 ± 18.45	49.63 ± 18.23	48.12 ± 16.92	< 0.001
Gender, n (%)						< 0.001
Male	9130 (48.61)	6246 (46.79)	1292 (50.16)	726 (54.92)	866 (56.49)	
Female	9651 (51.39)	7104 (53.21)	1284 (49.84)	596 (45.08)	667 (43.51)	
Race, n (%)						< 0.001
Non-white	10,628 (56.59)	7806 (58.47)	1367 (53.07)	675 (51.06)	780 (50.88)	
White	8153 (43.41)	5544 (41.53)	1209 (46.93)	647 (48.94)	753 (49.12)	
Smoking, n (%)						< 0.001
No	9463 (54.54)	6953 (57.31)	1201 (48.86)	619 (48.59)	690 (46.43)	
Yes	7887 (45.46)	5179 (42.69)	1257 (51.14)	655 (51.41)	796 (53.57)	
Education level, n (%)						< 0.001
Less than high school	5010 (29.15)	3226 (26.90)	829 (33.93)	437 (34.46)	518 (34.91)	
High school or above	12,177 (70.85)	8766 (73.10)	1614 (66.07)	831 (65.54)	966 (65.09)	
Body mass index, kg/m^2^	28.46 ± 6.68	27.75 ± 6.78	29.94 ± 6.26	30.29 ± 6.08	30.58 ± 5.78	< 0.001
Systolic blood pressure, mmHg	122.53 ± 19.03	121.15 ± 18.72	124.98 ± 19.31	126.52 ± 19.49	126.96 ± 19.23	< 0.001
Diastolic blood pressure, mmHg	68.88 ± 13.25	68.22 ± 12.88	70.26 ± 13.41	70.28 ± 14.15	71.12 ± 14.73	< 0.001
Energy, kcal	2149.28 ± 1020.81	2146.82 ± 1032.72	2112.49 ± 991.50	2144.78 ± 957.29	2237.06 ± 1015.15	0.003
eGFR, mg/min/1.73m^2^	92.49 ± 29.80	93.07 ± 28.43	90.16 ± 31.21	91.43 ± 34.66	92.23 ± 34.06	< 0.001
C-reactive protein, mg/L	0.45 ± 0.86	0.42 ± 0.87	0.51 ± 0.97	0.51 ± 0.66	0.53 ± 0.72	< 0.001
Triglycerides						
mg/dL	131.38 ± 87.24	89.44 ± 30.20	171.66 ± 14.46	222.04 ± 14.32	350.73 ± 107.34	< 0.001
mmol/L	1.48 ± 0.98	1.01 ± 0.34	1.94 ± 0.16	2.51 ± 0.16	3.96 ± 1.21	< 0.001
Low density lipoprotein cholesterol						
mg/dL	114.90 ± 35.92	111.57 ± 33.69	125.44 ± 37.33	125.36 ± 40.70	118.22 ± 44.07	< 0.001
mmol/L	2.97 ± 0.93	2.89 ± 0.87	3.24 ± 0.97	3.24 ± 1.05	3.06 ± 1.14	< 0.001
Total cholesterol						
mg/dL	194.24 ± 42.18	185.88 ± 38.09	207.80 ± 40.78	215.37 ± 44.77	226.00 ± 48.30	< 0.001
mmol/L	5.02 ± 1.09	4.81 ± 0.99	5.37 ± 1.05	5.57 ± 1.16	5.84 ± 1.25	< 0.001
High density lipoprotein cholesterol						
mg/dL	53.33 ± 15.65	56.43 ± 15.46	48.01 ± 13.17	45.95 ± 13.05	41.73 ± 12.96	< 0.001
mmol/L	1.38 ± 0.40	1.46 ± 0.40	1.24 ± 0.34	1.19 ± 0.34	1.08 ± 0.34	< 0.001
Diabetes, n (%)						< 0.001
No	15,898 (84.68)	11,739 (87.95)	2066 (80.26)	1006 (76.15)	1087 (70.91)	
Yes	2877 (15.32)	1608 (12.05)	508 (19.74)	315 (23.85)	446 (29.09)	
Hypertension, n (%)						< 0.001
No	11,387 (60.70)	8587 (64.38)	1384 (53.83)	678 (51.32)	738 (48.20)	
Yes	7374 (39.30)	4751 (35.62)	1187 (46.17)	643 (48.68)	793 (51.80)	
Cardiovascular disease, n (%)						< 0.001
No	15,712 (91.26)	11,076 (92.22)	2197 (89.64)	1118 (88.03)	1321 (88.96)	
Yes	1504 (8.74)	934 (7.78)	254 (10.36)	152 (11.97)	164 (11.04)	
Antihypertensive drugs, n (%)						< 0.001
No	14,358 (76.45)	10,557 (79.08)	1835 (71.23)	913 (69.06)	1053 (68.69)	
Yes	4423 (23.55)	2793 (20.92)	741 (28.77)	409 (30.94)	480 (31.31)	
Hypoglycemic agents, n (%)						< 0.001
No	17,309 (92.16)	12,516 (93.75)	2328 (90.37)	1167 (88.28)	1298 (84.67)	
Yes	1472 (7.84)	834 (6.25)	248 (9.63)	155 (11.72)	235 (15.33)	
Lipid-lowering drugs, n (%)						< 0.001
No	16,560 (88.17)	11,956 (89.56)	2193 (85.13)	1113 (84.19)	1298 (84.67)	
Yes	2221 (11.83)	1394 (10.44)	383 (14.87)	209 (15.81)	235 (15.33)	
Antiplatelet drugs, n (%)						< 0.001
No	18,460 (98.29)	13,153 (98.52)	2508 (97.36)	1292 (97.73)	1507 (98.30)	
Yes	321 (1.71)	197 (1.48)	68 (2.64)	30 (2.27)	26 (1.70)	
Cardiovascular mortality, n (%)						< 0.001
No	18,360 (97.76)	13,108 (98.19)	2493 (96.78)	1275 (96.44)	1484 (96.80)	
Yes	421 (2.24)	242 (1.81)	83 (3.22)	47 (3.56)	49 (3.20)	
All-cause mortality, n (%)						< 0.001
No	16,789 (89.39)	12,114 (90.74)	2243 (87.07)	1131 (85.55)	1301 (84.87)	
Yes	1992 (10.61)	1236 (9.26)	333 (12.93)	191 (14.45)	232 (15.13)	

Note: Subgroup differences were tested by one-way analysis of variance for continuous variables and by chi-square for categorical variables

Values are mean ± standardized differences or number (%)

*n* number; *eGFR* estimated glomerular filtration rate

### The relationship between triglyceride and mortality

As revealed in Table 2, for every 1 mmol/L increment in TG, TG (Model 3 HR = 1.08, 95%CI: 1.02, 1.15; *P* = 0.0085) was significantly associated with all-cause mortality, but not for cardiovascular mortality (Model 3 HR = 1.10, 95%CI: 0.97, 1.24; *P* = 0.1482). When using TG <  150 mg/dL as referent, the HRs for all-cause death were 0.97 (0.84, 1.12), 1.06 (0.89, 1.27) and 1.34 (1.12, 1.60) for TG levels at 150–200, 200–249 and ≥ 250 mg/dL in Model 3, respectively. (*P* for trend = 0.0070). Similarly, the HRs for death due to CVD were 1.01 (0.75, 1.37), 1.19 (0.82, 1.71) and 1.26 (0.85, 1.88) (*P* for trend = 0.2030), respectively.

**Table 2:** Multivariate Cox regression analysis of triglyceride levels with mortality in different models

	Model I HR (95%CI), *P*-value	Model II HR (95%CI), *P*-value	Model III HR (95%CI), *P*-value
All-cause mortality			
Triglycerides (per mmol/L increment)	1.17 (1.13, 1.21) < 0.0001	1.07 (1.02, 1.12) 0.0021	1.08 (1.02, 1.15) 0.0085
Triglycerides groups, mg/dL			
< 150	1.0	1.0	1.0
150–200	1.31 (1.16, 1.48) < 0.0001	1.00 (0.89, 1.13) 0.9414	0.97 (0.84, 1.12) 0.7249
200–249	1.39 (1.19, 1.62) < 0.0001	1.03 (0.88, 1.20) 0.7255	1.06 (0.89, 1.27) 0.5172
≥ 250	1.48 (1.28, 1.70) < 0.0001	1.32 (1.14, 1.51) 0.0001	1.34 (1.12, 1.60) 0.0016
P for trend	< 0.001	0.002	0.007
Cardiovascular mortality			
Triglycerides (per mmol/L increment)	1.22 (1.13, 1.31) < 0.0001	1.16 (1.06, 1.27) 0.0009	1.10 (0.97, 1.24) 0.1482
Triglycerides groups, mg/dL			
< 150	1.0	1.0	1.0
150–200	1.67 (1.30, 2.14) < 0.0001	1.28 (1.00, 1.65) 0.0504	1.01 (0.75, 1.37) 0.9349
200–249	1.77 (1.29, 2.41) 0.0004	1.31 (0.96, 1.79) 0.0909	1.19 (0.82, 1.71) 0.3544
≥ 250	1.61 (1.18, 2.18) 0.0025	1.53 (1.12, 2.08) 0.0068	1.26 (0.85, 1.88) 0.2517
P for trend	< 0.001	0.002	0.203

Notes: Multivariate Cox regression was performed to examine the association between triglyceride levels and mortality

Data are shown in HRs and 95%CI

*HR* hazard ratios; *CI* confidence intervals

Model I adjust for none

Model II adjust for age, gender and BMI

Model III adjust for age, gender, race, education level, smoking, body mass index, systolic blood pressure, estimated glomerular filtration rate, energy, C-reactive protein, total cholesterol, high density lipoprotein cholesterol, hypertension, diabetes, and medicine using (antihypertensive drugs, hypoglycemic agents, lipid-lowering drugs, and antiplatelet drugs)

**Fig. 3:**
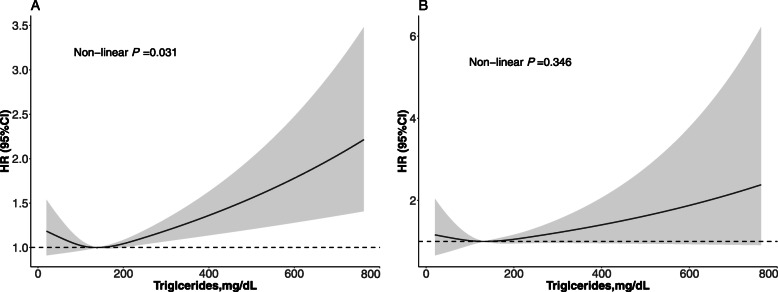
Adjusted spline curves analyze for the association of triglyceride with all-cause (**a**) and cardiovascular mortality. Age, gender, race, education level, smoking, body mass index, systolic blood pressure, estimated glomerular filtration rate, energy, C-reactive protein, total cholesterol, high density lipoprotein cholesterol, hypertension, diabetes, and medicine using (antihypertensive drugs, hypoglycemic agents, lipid-lowering drugs, and antiplatelet drugs)were all adjusted

As demonstrated in Table 3**,** the cut-off values of TG to predict death due to all-cause and cardiovascular diseases were 1.52 mmol/L (135 mg/dL) and 1.10 mmol/L (97 mg/dL), respectively. Below the cut-off value, the HRs for of all-cause and CVD death were 0.87 (95%CI: 0.71, 1.06) and 0.59 (95%CI: 0.25, 1.39) for every 1 mmol/L elevation in the level of serum TG. Above the cut-off value of TG levels, the HRs for of all-cause and CVD death were 1.12 (95%CI: 1.05, 1.19) and 1.12 (95%CI: 0.99, 1.27) for every 1 mmol/L elevation in the level of serum TG, respectively. The risk of death due to all-cause was the lowest when TG was approximately 135 mg/dL. Results from smoothing spline plots suggested that TG was linked with all-cause death in dose-independent manner (Fig. 3a), but it was close to a linear relationship with cardiovascular mortality (Fig. 3b).

**Table 3:** The results of two-piecewise linear regression model between triglyceride and mortality

	All-cause mortality HR (95% CI) *P*-value	Cardiovascular disease mortality HR (95% CI) *P*-value
Cutoff value, mmol/L	1.52 mmol/L (135 mg/dL)	1.10 mmol/L (97 mg/dL)
< Cut-off value	0.87 (0.71, 1.06) 0.1656	0.59 (0.25, 1.39) 0.2247
≥ Cut-off value	1.12 (1.05, 1.19) 0.0006	1.12 (0.99, 1.27) 0.0747
P for log likelihood ratio test	0.026	0.159

Notes: Multivariate linear regression was performed to examine the association between triglyceride levels and mortality

Data are shown in HRs and 95%CI

*HR* hazard ratios; *CI* confidence intervals

Effect: age, gender, race, education level, smoking, body mass index, systolic blood pressure, estimated glomerular filtration rate, energy, C-reactive protein, total cholesterol, high density lipoprotein cholesterol, hypertension, diabetes, and medicine using (antihypertensive drugs, hypoglycemic agents, lipid-lowering drugs, and antiplatelet drugs)

### Subgroup analysis

Table 4 has summarized the results of subgroup analysis. When TG ≥ 135 mg/dL, TG independently associated with all-cause death in subjects aged ≥65 years (HR: 1.09, 95%CI: 0.99, 1.19), males (HR: 1.14, 95%CI: 1.05, 1.23), people with BMI ≥ 25 kg/m^2^ (HR: 1.11, 95%CI: 1.03, 1.19),White population (HR: 1.16, 95%CI: 1.07, 1.27), people without taking lipid-lowering drugs (HR: 1.12, 95%CI: 1.05, 1.20), people without diabetes (HR: 1.11, 95%CI: 1.03, 1.19), people with hypertension (HR: 1.14, 95%CI: 1.06, 1.22), people with diabetes (HR: 1.11, 95%CI: 1.01, 1.22), and people with CVD (HR: 1.11, 95%CI: 1.03, 1.19). However, when TG was ≥97 mg/dL, TG associated with cardiovascular mortality in subjects with BMI < 25 kg/m^2^ (HR: 1.44, 95%CI: 1.09, 1.90), males (HR: 1.21, 95%CI: 1.05, 1.40), people with hypertension (HR: 1.25, 95%CI: 1.07, 1.46) and people with CVD (HR: 1.26, 95%CI: 1.04, 1.52).

**Table 4:** Subgroup analysis of triglycerides levels with mortality

	Number	All-cause mortality HR (95% CI) *P*-value		P for log likelihood ratio test	Cardiovascular disease mortality HR (95% CI) *P*-value		P for log likelihood ratio test
Cutoff value, mmol/L		< 1.52	≥ 1.52		< 1.10	≥ 1.10	
Age, years							
≥ 65	2502	0.78 (0.60, 1.01) 0.0558	1.09 (0.99, 1.19) 0.0654	0.024	0.54 (0.19, 1.51) 0.2400	1.00 (0.84, 1.19) 0.9968	0.267
< 65	9135	1.21 (0.87, 1.68) 0.2640	1.05 (0.95, 1.15) 0.3327	0.436	1.49 (0.30, 7.35) 0.6273	1.15 (0.96, 1.38) 0.1206	0.757
Gender							
Male	5659	0.91 (0.69, 1.19) 0.4874	1.14 (1.05, 1.23) 0.0016	0.136	0.85 (0.29, 2.50) 0.7651	1.21 (1.05, 1.40) 0.0096	0.527
Female	5978	0.89 (0.65, 1.22) 0.4596	1.08 (0.97, 1.20) 0.1491	0.277	0.44 (0.10, 2.00) 0.2873	0.92 (0.72, 1.18) 0.5239	0.367
Body mass index, kg/m^2^							
≥ 25	8102	0.90 (0.70, 1.16) 0.4312	1.11 (1.03, 1.19) 0.0036	0.143	0.70 (0.22, 2.19) 0.5437	1.09 (0.94, 1.26) 0.2783	0.471
< 25	3535	0.78 (0.54, 1.12) 0.1821	1.23 (1.03, 1.47) 0.0244	0.050	0.43 (0.11, 1.67) 0.2241	1.44 (1.09, 1.90) 0.0095	0.105
Race							
Non-white	6114	1.00 (0.74, 1.35) 0.9952	1.05 (0.96, 1.16) 0.2861	0.757	0.34 (0.10, 1.11) 0.0738	1.17 (0.98, 1.39) 0.0916	0.054
White	5523	0.79 (0.60, 1.05) 0.1088	1.16 (1.07, 1.27) 0.0007	0.016	1.08 (0.30, 3.96) 0.9055	1.07 (0.89, 1.27) 0.4852	0.982
Lipid-lowering drugs							
No	10,251	0.91 (0.73, 1.15) 0.4476	1.12 (1.05, 1.20) 0.0015	0.114	0.53 (0.20, 1.42) 0.2078	1.14 (0.98, 1.32) 0.0813	0.147
Yes	1386	0.75 (0.48, 1.17) 0.2035	1.11 (0.96, 1.29) 0.1696	0.126	1.05 (0.16, 6.85) 0.9565	1.07 (0.83, 1.37) 0.6183	0.990
Hypertension							
No	6779	0.87 (0.59, 1.29) 0.4962	1.07 (0.94, 1.23) 0.2792	0.350	2.16 (0.22, 21.49) 0.5106	0.95 (0.68, 1.34) 0.7752	0.483
Yes	4858	0.81 (0.64, 1.03) 0.0903	1.14 (1.06, 1.22) 0.0006	0.013	0.46 (0.18, 1.17) 0.1035	1.16 (1.01, 1.33) 0.0358	0.066
Diabetes							
No	9867	0.90 (0.71, 1.14) 0.3882	1.14 (1.04, 1.25) 0.0065	0.101	0.85 (0.30, 2.43) 0.7648	0.91 (0.72, 1.14) 0.4063	0.911
Yes	1770	0.82 (0.55, 1.22) 0.3276	1.11 (1.01, 1.22) 0.0287	0.162	0.43 (0.09, 2.17) 0.3091	1.25 (1.07, 1.46) 0.0054	0.223
CVD							
No	10,646	0.93 (0.74, 1.18) 0.5569	1.10 (1.02, 1.18) 0.0142	0.217	0.83 (0.28, 2.43) 0.7333	1.01 (0.85, 1.20) 0.8977	0.727
Yes	991	0.64 (0.42, 0.98) 0.0405	1.18 (1.04, 1.34) 0.0112	0.012	0.35 (0.07, 1.64) 0.1826	1.26 (1.04, 1.52) 0.0192	0.123

Notes: Multivariate Cox regression was performed to examine the association between triglyceride levels and mortality

Data are shown in HRs and 95%CI

*HR* hazard ratios; *CI* confidence intervals; *CAD* coronary heart disease

When analyzing a subgroup variable, age, gender, race, education level, smoking, body mass index, systolic blood pressure, estimated glomerular filtration rate, C-reactive protein, energy, total cholesterol, high density lipoprotein cholesterol, hypertension, diabetes, medicine use (antihypertensive drugs, hypoglycemic agents, lipid-lowering drugs, and antiplatelet drugs) were all adjusted except the variable itself

## Discussion

In this study, results showed that elevated TG had independent association with all-cause mortality. When compared to people with TG <  150 mg/dL, TG ≥ 250 mg/dL increased the risk of all-cause mortality increased by 34%.

How serum TG related to all-cause mortality in the present study agreed with some previous studies [2, 6, 7]. In this study, all-cause mortality risk was increased by 8% per 1 mmol/L TG increment. Meanwhile, the association between TG and CVD death did not reach significance level. A meta-analysis demonstrated that the risk of CVD death elevated by 13% (*P* <  0.001) per 1 mmol/L TG increment, suggesting TG associated with CVD mortality and was independent from multiple cardiovascular risk factors [7]. In addition, adjusted spline curves analyze showed when TG was greater than 135 mg/dL, the risk of all-cause mortality was elevated, suggesting a nonlinear relationship between TG and mortality. This finding was similar to previous studies [7, 17]. When TG <  135 mg/dL, TG was inversely associated with all-cause mortality. However, there was a study found that TG ranged 100 to 149 mg/dL might also increase the risk for mortality [18]. The differences in the study population may be the main reasons for the discrepancy in findings.

In the present study, the stratified analysis indicated no significant relationship between TG and cardiovascular mortality in all subgroups. However, the relationship was differed by age, gender, race, BMI, comorbidities, and the use of lipid-lowering drugs. Female participants have a lower risk of death than males, and could be explained by the role of estrogen. Previous research suggested that TG levels were significantly influenced by the endogenous hormonal environment [19–21]. It was also found that the relationship between TG and death was stronger among Whites, probably due to the difference in eating habits and genetic variations between ethnic groups.

The specific mechanism by which TG increased the risk of death has not been fully elucidated. First, animal and human experiments indicated that excessive TG levels were often accompanied by higher inflammation or oxidative stress [22, 23]. Second, circulating TG could passed the blood-brain and induce insulin receptor resistance [24]. Third, genetic variation of lipoprotein lipase, apolipoprotein C3 and lipase maturation factor-1 may play an important role [25]. Apolipoprotein C-III could associate with hypertriglyceridemia and CVD [26]. Although there were still many uncertainties in blood lipid metabolism, proprotein convertase subtilisin/kexin type 9 (PCSK9) has demonstrated an important role in lipid metabolism [27–29]. Moreover, high intrahepatic or circulating PCSK9 levels could increase TG storage and secretion, thus leading to a higher risk of CVD. These observations suggested the use of PCSK9 inhibitors to prevent CVD [30].

### Study strength and limitations

The strength of this study was to link with national data, which helped us to elucidate prospective relationship between TG and mortality. However, this study has some limitations. First, some covariates were self-reported. Second, there were other confounders not being adjusted, such as exercise and cardiovascular risk scores. Third, data on serum lipid was only collected once at baseline, and it was unclear how the changes in TG over time might influence the association with mortality. In addition, sample size was reduced due to incomplete data collection of serum lipids in NHANES. Finally, the study findings were mainly applicable to the American population and cannot be extrapolated to other countries.

## Conclusion

Elevated TG was independently associated with all-cause mortality, but no significant relationship with cardiovascular death. The results might also suggest non-linear correlation between TG and all-cause death. More attention should be paid to the association of TG with CVD-related mortality, and the management and monitoring of TG should be strengthened. In addition, the relationship between TG and cause-specific deaths is still unclear. More basic and clinical researches are still needed to clarify this relationship.

## Data Availability

Data are from the NHANES Study. Data are available in a public, open access repository.

## Abbreviations

CDC: Centers for Disease Control and Prevention
eGFR: Estimated glomerular filtration rate
CVD: Cardiovascular diseases
NHANES: National Health and Nutrition Examination Surveys
HR: Hazard ratio
CI: Confidence interval
TG: Triglyceride
LDL-C: Low density lipoprotein cholesterol
HDL-C: High density lipoprotein cholesterol
TC: Total cholesterol
SBP: Systolic blood pressure
DBP: Diastolic blood pressure
BMI: Body mass index
HbA1C: Hemoglobin A1C
